# Constitutional Chromothripsis on Chromosome 2: A Rare Case with Severe Presentation

**DOI:** 10.1155/2024/6319030

**Published:** 2024-01-30

**Authors:** Afia Hasnain, Laura L. Thompson, Nicole L. Hoppman, Karine Hovanes, Jing Liu, Bita Hashemi

**Affiliations:** ^1^Genomics Laboratory, Diagnostic Services, Shared Health, Winnipeg, MB, Canada; ^2^Division of Laboratory Genetics and Genomics, Department of Laboratory Medicine and Pathology, Mayo Clinic, Rochester, MN, USA; ^3^Natera, San Carlos, CA, USA; ^4^Genetics and Metabolism Program, Shared Health, Winnipeg, MB, Canada; ^5^Department of Pediatrics, Division of Genetics and Metabolism, Saskatchewan Health Authority, Saskatoon, SK, Canada

## Abstract

Chromothripsis is characterized by shattering and subsequent reassembly of chromosomes by DNA repair processes, which can give rise to a variety of congenital abnormalities and cancer. Constitutional chromothripsis is a rare occurrence, reported in children presenting with a wide range of birth defects. We present a case of a female child born with multiple major congenital abnormalities including severe microcephaly, ocular dysgenesis, heart defect, and imperforate anus. Chromosomal microarray and mate pair sequencing identified a complex chromosomal rearrangement involving the terminal end of the long arm of chromosome 2, with two duplications (located at 2p25.3-p25.1 and 2q35-q37.2 regions) and two deletions (located at 2q37.2-q37.3 and 2q37.3 regions) along with structural changes including inverted segments. A review of the literature for complex rearrangements on chromosome 2 revealed overlapping features; however, our patient had a significantly more severe phenotype which resulted in early death at the age of 2 years. Breakpoints analysis did not reveal the involvement of any candidate genes. We concluded that the complexity of the genomic rearrangement and the combined dosage/structural effect of these copy number variants are likely explanations for the severe presentation in our patient.

## 1. Background

Chromothripsis, a terminology first coined in patients with cancer [1], is defined as chromosomal shattering and random reassembly of fragments through various DNA damage repair mechanisms [2–6]. Soon after, constitutional chromothripsis was reported in several cases with a variety of phenotypes and chromosomal involvement [7–9]. In one case, transmission was reported from a balanced carrier parent [10], whereas one study reported stable segregation of this complex rearrangement in several family members [11]. The underlying mechanism of chromothripsis remains a matter of debate.

Single chromosomal involvement is rare in constitutional chromothripsis with currently only a few cases being reported [7, 12–14].

Terminal deletions and duplications of chromosome 2, as seen in our patient, are rare. Both are usually observed as part of more complex duplication/deletion syndromes. Rarely, terminal deletion of chromosome 2 has been reported in isolation and is known as chromosome 2q37 deletion syndrome. Reported features include hypotonia, brachycephaly, intellectual disability, autism spectrum disorder, short stature, obesity, minor facial dysmorphism, short neck, minor ear anomalies, congenital heart defects, nipple abnormalities, low anterior hairline, and low set ears [15]. To our knowledge, only a few patients have been reported with chromothripsis of chromosome 2, involving the chromosomal region of 2q34q37.2 [16, 17].

Determining the molecular and phenotypic consequences of genomic rearrangements is a major challenge, especially for patients with complex rearrangements. Genomic imbalances, position effects, or potential genes involved at or near breakpoints could be responsible for the clinical phenotype.

The aim of the present study was to characterize a complex rearrangement of chromosome 2, in a patient with severe congenital malformation involving the development of multiple major organs. We used mate pair sequencing in addition to the conventional techniques to explore the breakpoints and structural changes involved.

## 2. Case Presentation

We report a 2-year-old female born at term to a healthy 33-year-old G1P0 mother via uncomplicated normal vaginal delivery. The patient's family history was unremarkable. She was the first child of a nonconsanguineous couple of Filipino descent. Her maternal aunt passed away due to leukemia at 16 years of age. There was no history of congenital malformation or recurrent miscarriages in the couple or their relatives.

Prenatal history was unremarkable with no reported maternal illnesses or exposure to known teratogens. Prenatal ultrasound showed microcephaly and increased nuchal fold thickness. Maternal serum screening results were categorized as low risk for chromosomal aneuploidies and spina bifida. Parents decided to continue the pregnancy and declined any invasive testing.

At birth, growth parameters included a weight of 3610 g (>50^th^ centile), a length of 54.5 cm (95^th^ centile), and severe microcephaly with a head circumference of 29.5 cm (<2^nd^ centile). Physical examination of the patient at birth identified multiple dysmorphic features including a low anterior hairline, hypertelorism, short nose, depressed nasal bridge, left corneal opacity, right depressed globe, and bilateral simple low-set ears. She also had low-set nipples, bilateral supernumerary nipples in anterior axillary line, and a loud systolic murmur in cardiac examination. The anus was imperforated and anteriorly placed, while examination of external genitalia was typical for a normal female. She had a central and peripheral hypotonia with flexion contractures of elbows and camptodactyly of all fingers and adducted thumbs. She exhibited laryngomalacia.

Due to the patient's multiple congenital malformations, numerous clinical investigations were performed at birth. Her brain MRI revealed a thin corpus callosum with a dysplastic right orbit. Orbital ultrasound confirmed the anophthalmic socket on the right side. On the left side, the lens was contiguous with the cornea and the anterior chamber was flat, explaining the vascularized scar covering most of her left cornea. She failed her newborn hearing screen and was later confirmed to have bilateral moderate to severe sensorineural hearing loss. Her echocardiogram showed a small atrial septal defect with right to left shunt and a mild tricuspid regurgitation. She developed dilated cardiomyopathy with a moderate decrease in left ventricular function around 6 months of age. Abdominal and spinal ultrasounds were unremarkable.

At 3 months of age, she was admitted for a generalized tonic clonic seizure and was initiated on phenobarbital. During this admission, due to her relatively coarse facial features, urine glycosaminoglycan and oligosaccharide testing were performed, and the results came back normal. Her creatine kinase (CK) level was initially high at 5000 U/L, but subsequently normalized.

The patient underwent several corrective repair surgeries for imperforate anus and had a corneal graft in her left eye and right eye prosthesis. With continuing feeding difficulty, she required a surgery for the placement of a gastrostomy tube.

At 1 year of age, she had a severe global developmental delay with absent head control and only occasional cooing. She exhibited progressive microcephaly and developed severe brachycephaly.

The proband passed away at 2 years of age at home due to her multiple comorbidities.

Subsequently, the couple had a second pregnancy, resulting in a healthy daughter with normal development assessed at 6 months of age. The parents have provided consent for the publication of clinical and laboratory data.

## 3. Investigations

Single nucleotide polymorphism (SNP) array analysis was performed on the proband at birth in 2016 using a custom-designed Illumina SNP array at CombiMatrix/Invitae Corporation (Irvine, CA 92618), and the results were described relative to the reference genome (GRCh37/hg19). FISH analysis was performed at CombiMatrix/Invitae Corporation (Irvine, CA 92618) using probes RP11-60A11 (2p25.3), RP11-94I20 (2q37.1), RP11-526L8 (proximal 2q37.3) and RP11-875C22 (distal 2q37.3). Mate pair sequencing (MPSeq) was performed at the Mayo Clinic (Rochester, MN) as described previously [18, 19]. MPSeq data were analyzed with SVA tools for the detection of structural rearrangements or copy number variants relative to the reference genome (GRCh38/hg38) and visualized using in-house developed software. The breakpoints and junctions were reviewed for gene interruption and/or gene fusions using UCSC Genome Browser (GRCh38/hg38) [20], and CNVs were curated and analyzed for the number of genes, disease association, inheritance pattern, and corresponding triplosensitivity and haploinsufficiency scores using DECIPHER (DatabasE of genomiC varIation and Phenotype in Humans using Ensembl Resources), ClinVar, OMIM (Online Mendelian Inheritance in Man), DGV (Database of Genomic Variants), and ClinGen Dosage Sensitivity Curation databases [21–25].

## 4. Results

**Figure 1:**
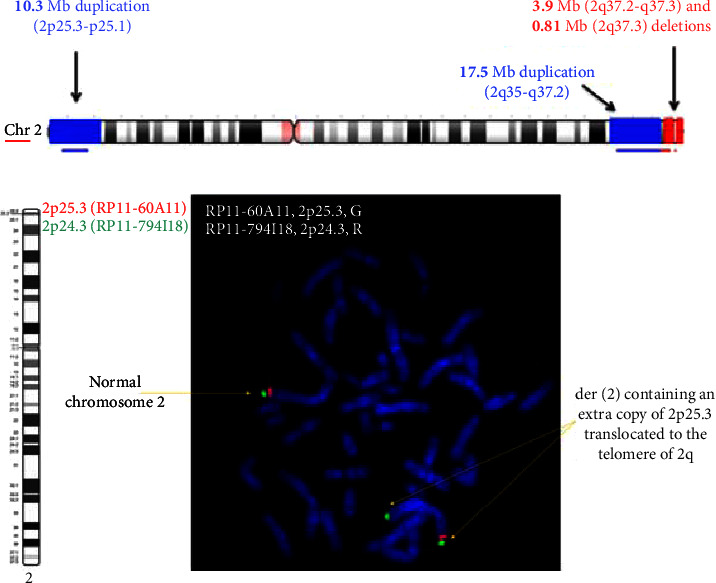
(a) Four CNVs were detected on chromosome 2: the two duplications which encompass the 2p25.3-p25.1 region (10.3 megabases (Mb) in size) and the 2q35-q37.2 (17.5 Mb) region and the two deletions which encompass the 2q37.2-q37.3 (3.9 Mb) region and the 2q37.3 (0.81 Mb) region. arr (GRCh37) 2p25.3p25.1 (0_10322310) × 3, 2q35q37.2 (219436155_236910276) × 3, 2q37.2q37.3 (236910276_240803999) × 1, and 2q37.3 (242397618_243199373) × 1. (b) Metaphase FISH analysis using probes for the four CNV regions confirmed the microarray findings. The duplication encompassing the 2p25.3-p25.1 region was found to be translocated to the terminal *q*-arm region of chromosome 2.

SNP microarray analysis identified a total of four copy number variants (CNVs) with a total of 5 breakpoints on chromosome 2: the two duplications that encompass the 2p25.3-p25.1 region (10.3 megabases (Mb) in size) and the 2q35-q37.2 (17.5 Mb) region and the two deletions which encompass the 2q37.2-q37.3 (3.9 Mb) region and the 2q37.3 (0.81 Mb) region. There was an intact region of approximately 1.6 Mb in size between the two deletions (Figure 1(a)). Metaphase FISH analysis confirmed these CNVs and showed that the 2p25.3 duplication is translocated to the telomeric region of 2q (Figure 1(b)). MPSeq analysis confirmed this translocation and showed that the duplication in the 2q35-q37.2 region was a tandem inverted duplication. The analysis further identified the intact 2q37.3 region between the two deleted segments as inverted in orientation.

Parental FISH testing and microarray performed on the couple's second, clinically unaffected child was normal, supporting the *de novo* occurrence of this complex chromosomal abnormality in the proband.

## 5. Discussion

Constitutional chromothripsis is a rare event and mostly *de novo* in origin. Most reported cases have two or more chromosomes involved in the complex chromosomal rearrangement. To our knowledge, only a few patients have been reported with chromothripsis implicating a single chromosome.

Chromothriptic events involving chromosome 2 have been reported previously in two cases with fewer breakpoints and smaller CNVs resulting in milder phenotypes including growth retardation and intellectual disability [16, 26].

In this study, we utilized SNP array to detect CNVs and MPSeq to detect the balanced and unbalanced structural rearrangements at a higher breakpoint resolution in our patient with *de novo* complex rearrangement of chromosome 2.

**Figure 2:**
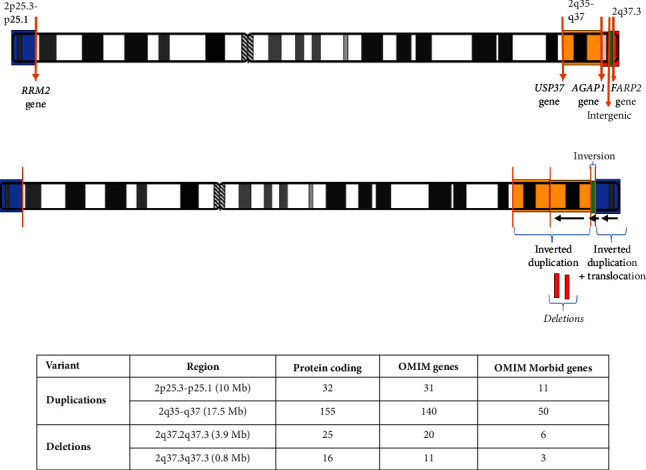
The schematic diagram of the normal and derivative of chromosome 2 with breakpoints and structural and copy number variants. (a) Normal chromosome 2: The breakpoints were reviewed for gene disruption; 4 out of 5 breakpoints interrupted intronic regions of different genes not associated with a known disease phenotype at the present time. One of the breakpoints interrupted the intergenic region between NDUFA10 and LOC150935 long noncoding RNA genes. (b) The derivative of chromosome 2: The CNVs and inversions on the structurally rearranged chromosome 2. The two duplicated segments were found to be in an inverted orientation (black arrows), one of them was an inverted translocation from the *p*-terminal to the *q*-terminal region (blue segment 2p25.3-p25.1 region) and the other was a tandem inverted duplication (yellow segment 2q35-q37). Two deletions (red segments 2q37.2q37.3 and 2q37.3q37.3) were separated by an inverted copy neutral region (green segment 2q37.3 region). (c) The CNVs were reviewed for the number of protein-coding genes, OMIM genes, and OMIM Morbid genes. No triplosensitive or haploinsufficient genes were found in the duplicated and deleted regions, respectively.

The five breakpoints on chromosome 2 resulted in two inverted duplications, two deletions, and one inversion (Figure 2(b)). To interpret this complex structural rearrangement in the context of this patient's severe phenotype, we closely examined and reviewed each breakpoint and CNV in DECIPHER, ClinVar, OMIM, DGV, and ClinGen databases.

The two duplicated regions harbored 187 protein coding genes in total, 171 of which were OMIM genes and 61 were OMIM Morbid genes. None of the duplicated genes in these regions were found to be associated with triplosensitivity, in which an additional copy of a gene produces a phenotype. The two deleted regions contained 41 protein coding genes in total, 31 of which were OMIM genes and 9 were OMIM Morbid genes. None of these genes have established haploinsufficiency, in which loss of one copy of a gene could be responsible for the phenotype (Figure 2(c)). One of the deleted regions in our patient involves the chromosome 2q37 deletion syndrome (OMIM # 600430) region.

Four breakpoints interrupted the intronic regions of AGAP1, USP37, FARP2, and RRM2 genes, none of which is associated with a known disease phenotype at the present time (Figure 2(a)). One of the breakpoints fell within the intergenic region between NDUFA10 and LOC150935 long noncoding RNA gene. No gene fusions were detected.

For the phenotypic characterization of our patient, previous reports of cases with complex chromosome 2 rearrangements and associated syndromes within the region were reviewed. A summary of the clinical presentation among patients with 2q37 syndrome (OMIM # 600430), and case reports on complex 2p25 duplication and 2q37 deletion [16, 17, 26] with a comparison to our patient phenotype, is presented in Supplementary Table 1.

While we found a significant overlap of features among patients with a combination of deletion and duplication in the region, our patient had a far more severe phenotype with some unique features like imperforate anus, supernumerary nipples, and severe eye deformities not previously described with chromosome 2-related syndromes. The cumulative effect of this complex chromosomal rearrangement with CNVs, involving over 500 genes including 228 protein-coding genes, is likely responsible for the severe phenotype in this patient.

In cases with complex chromosomal rearrangements, a clear genotype-phenotype correlation is difficult to establish, as there is a potential for alterations involving gene regulation or chromatin structure that can disturb the gene expression and contribute to abnormal development. A future direction for research is to study the role of chromatin structures in gene expression in cases with complex rearrangements.

## Data Availability

The data that support the findings of this study are available from the corresponding author upon reasonable request.

## Supplementary Materials

Supplementary Materials Table 1: Comparison of phenotypic features of case reports with 2p25 duplication and 2q37 deletion with our patient. Figure 1: Mate pair sequencing results.
