# Supplementary material for: Constitutional Chromothripsis on Chromosome 2: A Rare Case with Severe Presentation
Source: Case Rep Genet. 2024 Jan 30;2024:6319030. doi: 10.1155/2024/6319030 (PMC10846923; doi:10.1155/2024/6319030)
Supplement: Supplementary Materials — Table 1: Comparison of phenotypic features of case reports with 2p25 duplication and 2q37 deletion with our patient. Figure 1: Mate pair sequencing results. [file 6319030.f1.zip › MPSeq Results for Supplemental Figure with legend.docx]

# A B


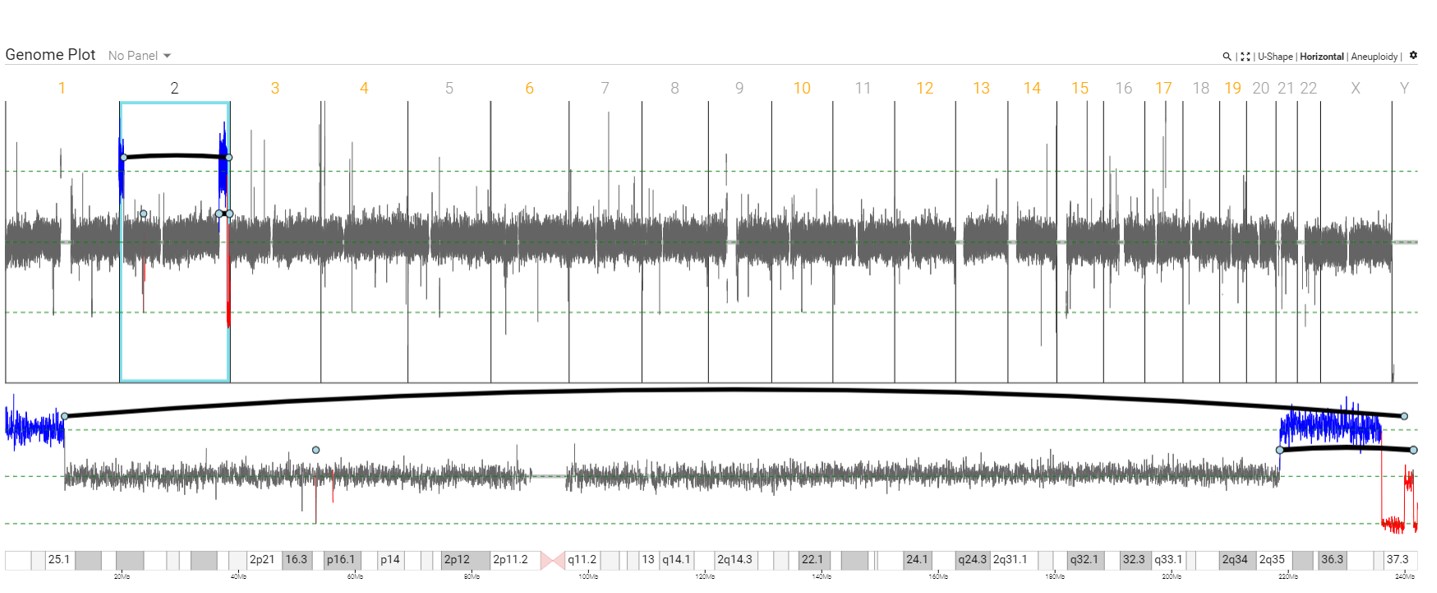

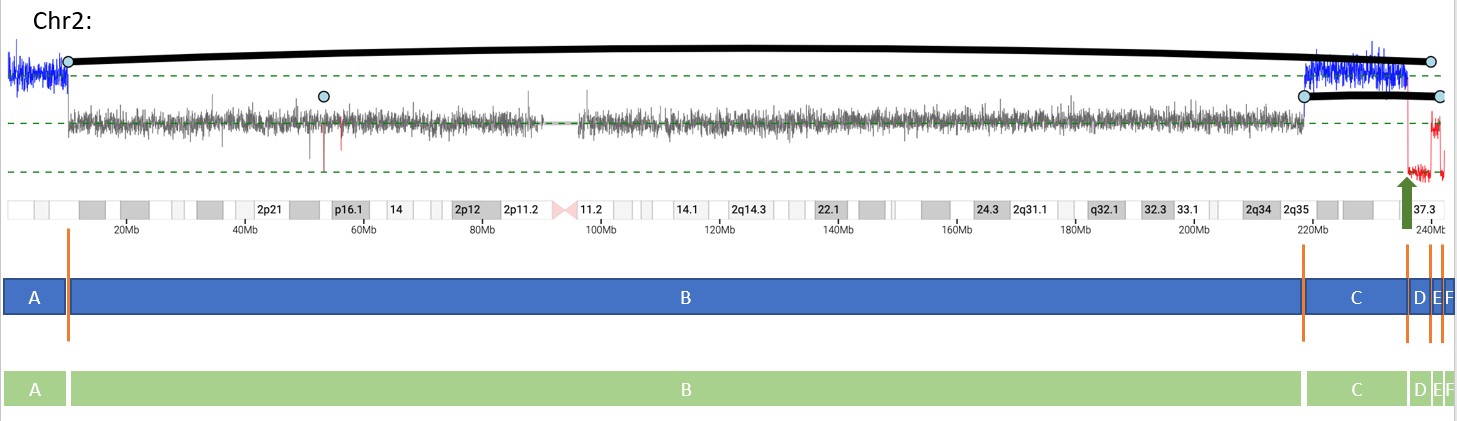


# D


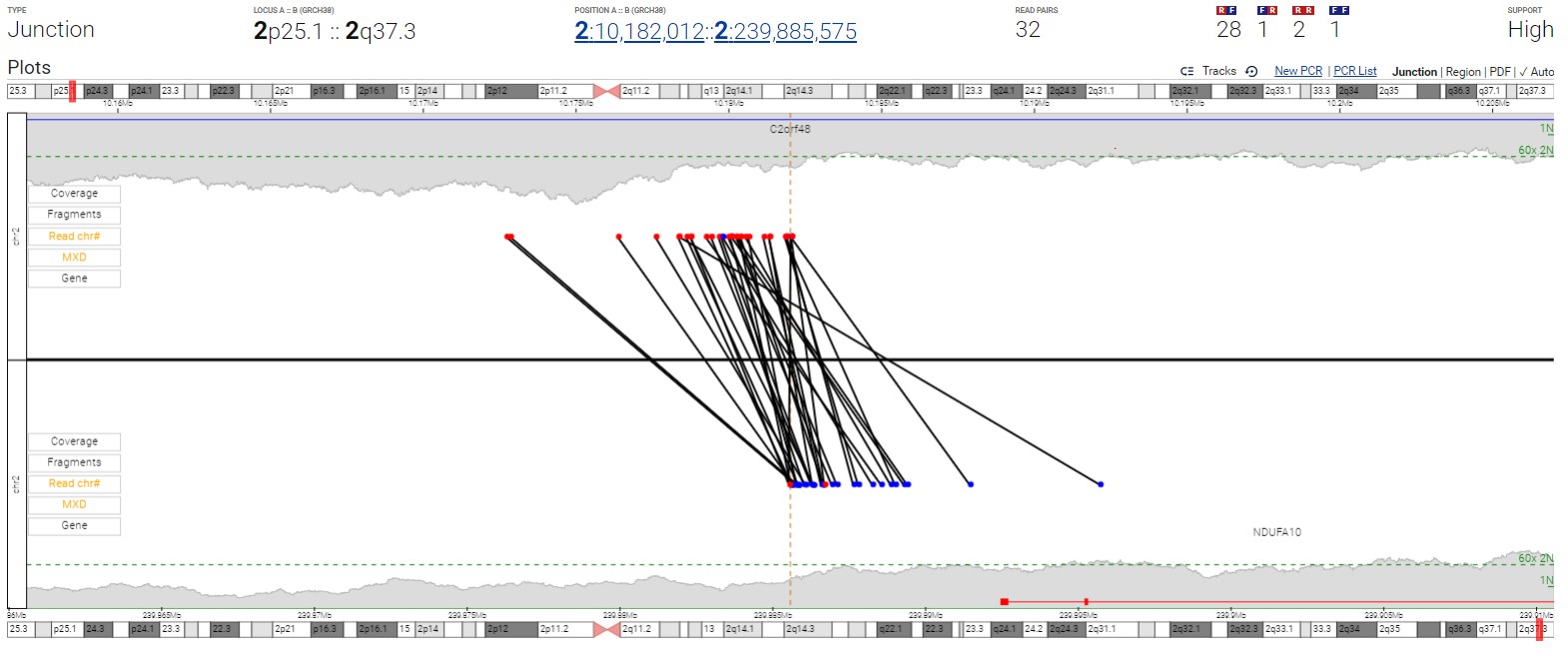

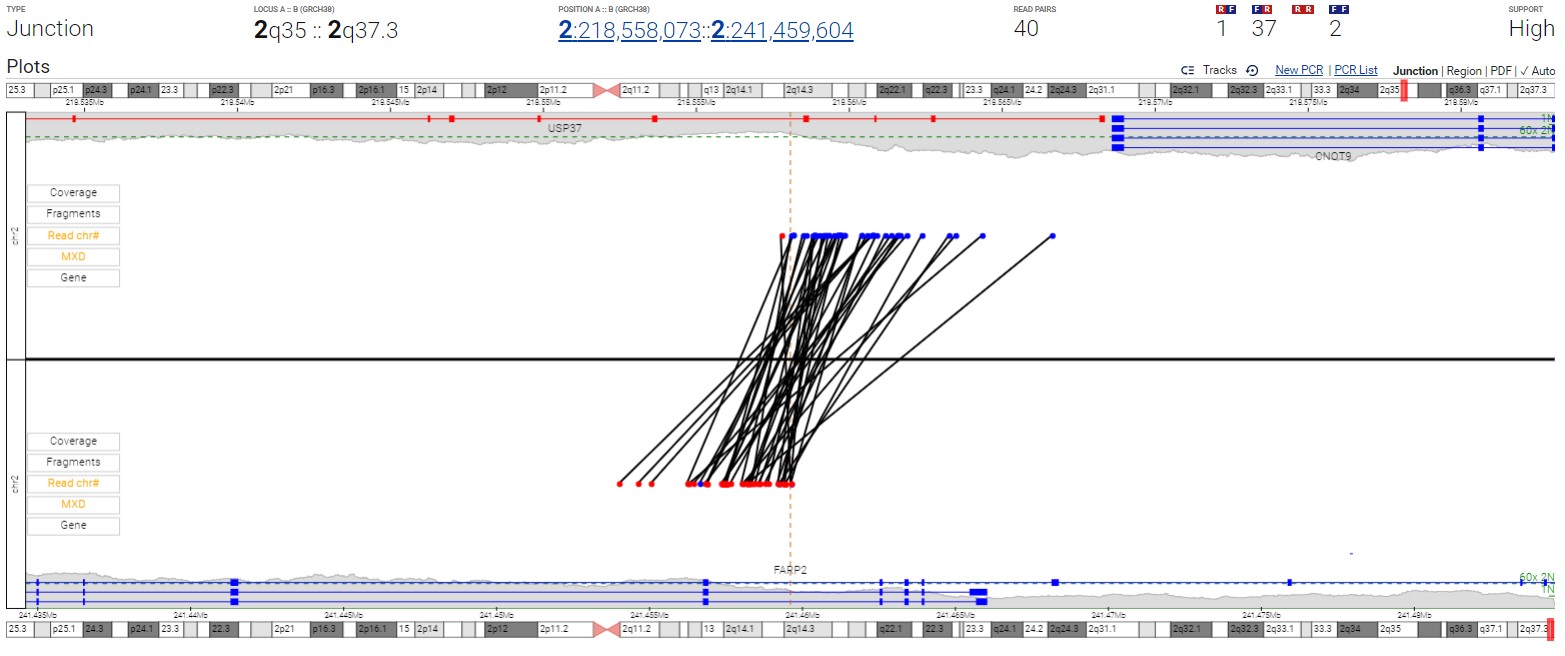


Suppl Fig 1: Mate-pair sequencing results. A) Genome-wide view at the top where Chromosome 2 is selected showing the copy number reads for chr2. Blue indicates gains, red losses, and grey indicates an expected copy number of 2. B) Chromosome 2 map showing gains (blue) and losses (red). C and D) The junction plot view. Ideograms at top and bottom indicate where the reads are mapping to. Forward read (red) mapping with a reverse read (blue).
