# Supplementary material for: Constitutional Chromothripsis on Chromosome 2: A Rare Case with Severe Presentation
Source: Case Rep Genet. 2024 Jan 30;2024:6319030. doi: 10.1155/2024/6319030 (PMC10846923; doi:10.1155/2024/6319030)
Supplement: Supplementary Materials — Table 1: Comparison of phenotypic features of case reports with 2p25 duplication and 2q37 deletion with our patient. Figure 1: Mate pair sequencing results. [file 6319030.f1.zip › Supplementary Table 1 (1) (1).docx]

|  | **Patient in this case study** | **2q37 syndrome** | **2p25 duplication and 2q37**  **deletion**  **PMID: 24963**  **351** | **Patient 1 duplication of 2p23 and deletion of 2q37 in 2 unrelated patients**  **PMID: 15742**  **366** | **Patient 2 duplication of 2p23 and deletion of 2q37 in 2 unrelated patients**  **PMID: 15742**  **366** | **1.9Mb duplication in the**  **chromosom**  **al region**  **2q37.2 and a5.4Mb deletion on chromosom**  **e 2q37.3** | **2q37.3 del and 2q34q37.2 dup Hetergeneit y of CCR in**  **2q** |
| --- | --- | --- | --- | --- | --- | --- | --- |
|  |  | **OMIM # 600430** | **PMID:**  **24963351** | **PMID:**  **15742366** | **PMID:**  **15742366** | **PMID:**  **26822876** | **PMID:**  **27216161** |
| **Chromosome rearrangement** | arr[GRCh37]2p25.3p25.1(0_1032 2310)x3,2q35q37.2(219436155_2  36910276)x3,2q37.2q37.3(236910  276_240803999)x1,2q37.3(24239  7618_243199373)x1 |  | arr[hg19] 2p25.3p25.1  (30,3419,588,369)x3  ,2q37.2q37.3 (235,744,424  -  243,041,305) x1 | dup 2p23 & del 2q37 | dup 2p23 & del 2q37 | dup 2q37.2 and del 2q37.3 |  |
| **Age at diagnosis** | Prenatal | birth | 4 months | birth | 8 months | prenatal femoral shortening | prenatal |
| **Sex** | Female |  | male | male | female | female | female |
| **inheritance** | De novo |  | de no vo | inherited pericentric maternal inversion | de novo | de novo | de novo |
| **Number of SVs** | **5** |  | 2 | 2 | 2 | 2 | 17 |
| **Growth** | Short stature |  | + |  |  |  |  |
| **Head** | Brachycephaly | + | + |  |  |  |  |
|  | Microcephaly |  | + |  |  |  |  |

|  | Scaphocephaly |  |  |  |  |  |  |
| --- | --- | --- | --- | --- | --- | --- | --- |
| **Face** | Coarse facial appearance | + |  |  |  |  |  |
|  | Midface hypoplasia | + |  |  |  |  |  |
|  | Low anterior hairline |  |  |  |  |  |  |
|  | Fleshy soft philtrum |  |  |  |  |  |  |
| **Eyes** | Hypertelorism |  |  |  |  |  |  |
|  | Corneal opacity |  |  |  |  |  | extropia |
|  | Depressed globe |  |  |  |  |  |  |
|  | Dysplastic right orbit |  |  |  |  |  |  |
|  | Anophthalmic socket |  |  |  |  |  |  |
|  | Epithelialized left cornea |  |  |  |  |  |  |
| **Nose** | Short nose |  |  |  |  | + |  |
|  | Depressed nasal bridge | + |  |  |  |  | + |
| **Teeth** | No teeth |  |  |  |  |  |  |
| **Ears** | Severe bilateral sensorineural hearing loss | + |  |  |  |  |  |
|  | Low set ears |  | + | + | + | + | + |
|  | Protruding simple ears |  |  |  |  |  |  |
|  | Large ears |  |  |  |  |  |  |
| **Cardiovascular** | Atrial septal defect | other | mitral valve  dysplasia |  |  | aortic insufficiency |  |
|  | Mild tricuspid regurgitation |  |  |  |  |  |  |
|  | Dilated cardiomyopathy |  |  |  |  |  |  |
| **SKELETAL** | Bilateral clinodactyly |  |  | + | + |  |  |
|  | Contractures of elbow |  |  |  |  |  |  |
|  | Camptodactyly of all fingers |  |  |  |  |  |  |
|  | Adducted thumbs |  |  |  |  |  |  |
|  | Hyperconvex nails |  |  |  |  |  |  |
|  | Long fingers and toes |  |  | + |  | + |  |
|  | Hyperextensible joints |  |  |  |  |  |  |
| **NEUROLOGIC** | Tonic-clonic seizure | + |  |  |  |  |  |
|  | Global developmental delay | + |  | + | + |  |  |
|  | Hypotonia |  |  | + | + |  |  |
|  | Thin corpus collosum |  |  |  |  |  |  |
|  | Absent speech |  |  |  |  | + |  |
|  | Low set nipples |  |  | abnormal | abnormal |  | transverse fold under  nipples |
|  | Supernumerary nipple |  |  |  |  |  |  |
|  | Imperforate anus |  |  |  |  |  |  |
| **Others** | Perianal fistula |  |  |  |  |  |  |
|  | Laryngomalacia |  |  |  |  |  |  |
|  | Sacral dimple |  |  | + | + |  | + |

**Supplementary Table 1:** A summary of the clinical presentation among patients with 2q37 syndrome (OMIM # 600430), and case reports on complex 2p25 duplication and 2q37 deletion with a comparison to our patient’s phenotype. The severe phenotype in our patient that is not previously reported in the literature is highlighted in blue.
